# “Periodontal ligament‐on‐chip” as a Novel Tool for Studies on the Physiology and Pathology of Periodontal Tissues

**DOI:** 10.1002/adhm.202303942

**Published:** 2024-09-16

**Authors:** Sara Svanberg, Elisabeth Hirth, Thimios A. Mitsiadis, Petra S. Dittrich

**Affiliations:** ^1^ ETH Zürich Schanzenstrasse 44 Basel 4056 Switzerland; ^2^ Institute of Oral Biology University of Zurich Plattenstrasse 11 Zürich 8032 Switzerland

**Keywords:** HUVECs, lipopolysaccharides, periodontal ligament cells, “periodontal ligament‐on‐chip”, periodontitis, vascularization

## Abstract

Teeth exert fundamental physiological functions, such as mastication and speech, and are a key feature of oral health that affects life quality. Teeth are anchored to the alveolar bone via the periodontal ligament, which provides stability to the teeth and absorbs mechanical stresses during mastication. Periodontal infection leads to periodontitis, a severe inflammation of the supporting soft tissues that ultimately cause tooth loss. Despite the pressing need of periodontal regeneration for improved oral care, efficient in vitro models of the periodontal tissues are still missing, thus hampering the development of novel, faster, and more effective therapy modalities. Herein, a novel “periodontal ligament (PDL)‐on‐chip” model that integrates patient‐derived periodontal ligament cells (PDLCs) and endothelial cells is introduced. This microfluidic platform provides optimal conditions for the formation of extensive and perfusable vascular networks. Furthermore, PDLCs elicit blood vessels’ development and maturation while establishing close contacts with the endothelial cells. Potential applications for inflammatory periodontal diseases are also successfully displayed in the “PDL‐on‐chip” by stimulating inflammation and detecting inflammatory cytokines. This work offers a cornerstone for more complex and specialized microfluidic dental models, which are necessary to unravel complex oral diseases that affect individuals’ general health that go beyond the field of dentistry.

## Graphical abstract

Teeth are crucial for oral health and well‐being. Periodontal infections like periodontitis often result in tooth loss. Addressing the lack of effective in vitro models, a microfluidic model of the vascularized periodontal ligament tissue is created. The model successfully simulates inflammation and marks a significant step toward understanding complex oral health issues beyond traditional dentistry.

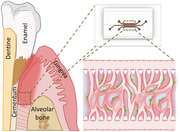

## 1 Introduction

Periodontium is a specialized heterogenous tissue supporting the tooth and is composed by the tooth root cementum, the periodontal ligament (PDL) and the alveolar bone.^[^ ^1^ ^]^ PDL is a highly vascularized and innervated connective soft tissue that is situated between the alveolar bone and the cementum and serves for tooth stability and functions such as chewing, biting, and speech. PDL is a dynamic environment under constant remodulation and regeneration due to the mechanical stresses during mastication.^[^ ^2^, ^3^, ^4^ ^]^ PDL is a common site of pathologies that severely affect not only the structures of the dental root cementum and alveolar bone, but also tooth functionality that has tremendous impact on well‐being, self‐esteem, and social situation. Inflammatory periodontal diseases are among the most common chronic infection diseases and are affecting 20–40% of the global population.^[^ ^5^, ^6^ ^]^ These infectious diseases get more prone with age and therefore constitute a significant global health concern within the constantly increasing aging population.^[^ ^7^, ^8^ ^]^ Periodontitis is the most severe periodontal disease, resulting in degradation of the gum and anchoring bone that most frequently causes tooth loss.^[^ ^3^, ^9^ ^]^

Periodontitis can develop upon traumatic tooth injury, infectious and genetic diseases, but the primary cause of its development is the neglected oral hygiene that allows the formation of microbial plaques on the tooth surfaces. The resident microbes trigger an immunological host response, resulting in destruction of the hard tissues of the periodontium.^[^ ^10^ ^]^ Periodontitis does not affect only the periodontal tissues but also the tissues of other vital organs, thus playing an important role in the prognosis of systemic diseases, including cardiovascular diseases, diabetes, Alzheimer's disease, adverse pregnancies, and respiratory tract infections.^[^ ^11^ ^]^ This link between periodontitis and systemic diseases is still not fully studied and understood. It is believed (hypothesized) that the presence of periodontium‐derived microbes and their toxins and metabolic products could modulate the immune responses, which promote systemic conditions development.^[^ ^12^ ^]^ It has been suggested that lipopolysaccharides (LPS), endotoxins located in the outer membrane of gram‐negative bacteria, play a crucial role in the progression of periodontitis. Gram‐negative bacteria are usually enriched during oral dysbiosis in periodontitis. It is believed that the LPS endotoxins infiltrate into the periodontal tissue, where through its rich vascular network could reach and also affect the systemic circulation (endotoxemia).^[^ ^13^ ^]^ Thereby, LPS endotoxins can cause both local and systemic inflammation by activating both innate and adaptive immunity.^[^ ^14^ ^]^

Current treatments of periodontitis rely on deep cleaning, antibiotics, and surgery in severe cases.^[^ ^2^, ^3^, ^15^ ^]^ Periodontitis is a multifactorial disease and efficient treatment modalities cannot be developed until it will be a thorough understanding of the molecular and cellular processes underlying the physiology and pathology of the periodontal tissues. Hence, this gap in research has attracted attention from other medical fields beyond dentistry. Despite the extreme medical significance of periodontitis, we are still lacking efficient in vitro models that emulate the physiopathology of dental tissues, including the periodontal ligaments. It is therefore obvious that the development of innovative in vitro models simulating the functions of the human periodontal ligaments is of central importance.

“Organ‐on‐chip” technologies have become useful tools in recapitulating complex human tissues by mimicking tissue‐specific cellular microenvironments. Microfluidic platforms enable precise spatio‐temporal control of a given tissue and thereby serve as advantageous tools compared to simplistic 2D cultures. In addition, these 3D devices prevent the use of animal models, which often fail to replicate the human physiological responses.^[^ ^16^ ^]^ Vast “organ‐on‐chip” models have already been established^[^ ^17^ ^]^ such as “lung‐on‐chip”,^[^ ^18^ ^]^ “intestine‐on‐chip”,^[^ ^19^ ^]^ “bone‐on‐a‐chip”,^[^ ^20^ ^]^ and “kidney‐on‐chip”.^[^ ^21^ ^]^ Up to date we even have several generations of “organ‐on‐chip” models^[^ ^22^ ^]^ or even coupled organs representing the “full body‐on‐chip”.^[^ ^23^ ^]^ Despite the rich plethora of “organ‐on‐chip” models, dental tissues such as the periodontal ligaments, have remained relatively unexplored.^[^ ^24^ ^]^

Microfluidics has been employed for dental models.^[^ ^25^, ^26^, ^27^, ^28^, ^29^, ^30^, ^31^, ^32^, ^33^ ^]^ For example, Franca et al.^[^ ^29^ ^]^ developed a “tooth‐on‐chip” platform to study dental stem cell response to biomaterials. Later, Rodrigues et al.^[^ ^30^ ^]^ developed a platform to model the biomaterial‐biofilm‐dentine interface. Thereafter, Dhall et al.^[^ ^31^ ^]^ developed a “dental implant‐on‐chip” model that was challenged with streptococcus mutans to study host‐material‐pathogen interaction. Most recently, Makkar et al.^[^ ^32^ ^]^ presented a platform for modeling crevicular fluid flow and host‐oral microbiome interaction in a “gingival crevice‐on‐chip” and Muniraj et al.^[^ ^33^ ^]^ modeled the gingival epithelial barrier using a full‐thickness gingiva‐on‐chip to study ulcers. However, neither of these models recapitulated the periodontal ligaments including perfusable vasculature and complex 3D structures toward unraveling the underlying pathophysiology of periodontal disease. Dental tissues, including the periodontal ligaments, are highly vascularized. The soft tissue of periodontal ligaments consists of 20% vascular volume compared to 5% found in most other connective tissues in the body.^[^ ^34^ ^]^ Furthermore, vasculature is involved in fundamental physiological processes and is associated with various diseases and their progression. The vasculature is also a site for early immunological responses.^[^ ^35^ ^]^ Hence, it is crucial to incorporate vasculature in dental models for studying pathophysiological events and developing drug screening platforms.^[^ ^36^, ^37^ ^]^

Here, we used a microfluidic approach to create a novel 3D in vitro vascularized periodontal ligament model that emulates the physiopathology of human periodontal ligament tissue. This model consists of human‐derived periodontal ligaments cells (PDLCs) and endothelial cells, which form a vascular perfusable network. Fibrin hydrogel is used as a scaffold to create the complex 3D microenvironment of the periodontal ligaments. Furthermore, as a proof of concept, we demonstrated the relevance of the model for studying early stages of inflammatory diseases. We emulated inflammation in our model by adding LPS endotoxins and subsequently monitored secretion of cytokines and characterized extracellular matrix proteins and inflammatory markers. These findings constitute an important foundation for creating complex models of dental tissues in order to better understand the tooth‐specific inflammatory diseases and to imagine novel techniques for their treatments. Overall, this “PDL‐on‐chip” model represents a physiologically relevant model of the highly vascularized human soft periodontal ligament tissue for simulating early stages of periodontitis and constitute a great promise for improved dental care in the future.

## 2 Results

### 2.1 Chip Design and Operation

**Figure 1:**
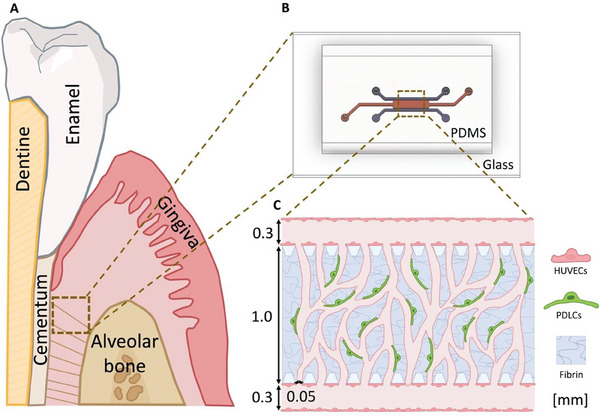
Sketch of periodontal tissue and the microvasculature emulated on a microfluidic device. A) Cross‐section of the tooth with periodontal ligaments, the tissue of interest, marked in a box. B) The microfluidic three channel design used to cultivate cells. C) The tissue formed on the microfluidic device shows an inter‐connected vascular network in co‐culture with periodontal ligament cells (PDLCs) supported by a fibrin hydrogel scaffold.

**Figure 2:**
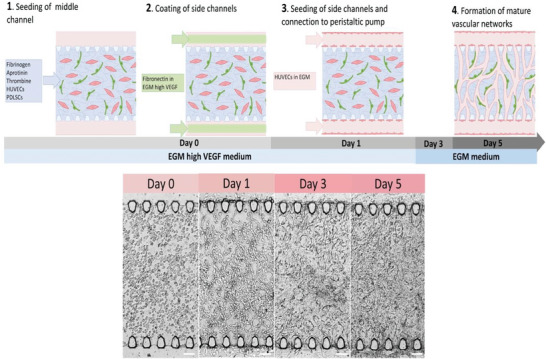
Schematic overview of the cell seeding procedure and the resulting cell growth on the microfluidic chip that is being medium perfused from a peristaltic pump over five days. The first step in creating PDL‐on‐chip is to embed HUVECs and PDLCs in a fibrin hydrogel. The next day, the cells are transformed from a round shape into more elongated shapes and the side channels are seeded with a monolayer of HUVECs. Thereafter, the chips are connected to a peristaltic pump and perfused with medium at 60 µL h^−1^. On day 3, a vascular network can already be observed. On day 5, the vascular network is more mature and connected with the side channels. Microfluidic chips for static cultivation were seeded in the same manner but were cultured with medium filled pipette tips with daily exchange instead of being connected to peristaltic pump. Scale bars: 100 µm.

We employed a microfluidic device with three parallel channels separated by micropillars to recapitulate the highly vascularized periodontal ligaments (**Figure** **1**). A silicon wafer was fabricated as master mold using standard photolithography methods. The device is made of polydimethylsiloxane (PDMS) because of its biocompatible and transparent properties and is furthermore bonded to glass. The middle channel (length 4400 µm, width 1000 µm, and height 100 µm) is loaded with a fibrin hydrogel containing a co‐culture of red fluorescent protein (RFP) producing human umbilical vein cells (HUVECs) and patient‐derived periodontal ligament cells (PDLCs) in a 5:1 ratio. The fibrin hydrogel is confined between the micropillars owing to hydrophobic forces. The side channels (width 300 µm and height 100 µm) are seeded with a monolayer of HUVECs on the hydrogel interface to improve the connectivity between the fibrin embedded vasculature and the side channels (**Figure** **2**).

The cells on chip are either cultured statically with pipette tips as medium reservoirs or cultured under continuous medium perfusion at 60 µL h^−1^ (corresponding to 0.33 dyne cm^−2^) using a peristaltic pump (Culture methods/set ups in Figure S1, Supporting Information, bright‐field images in Figure S2, Supporting Information). Continuous perfusion ensures that medium is constantly replenished while waste products are being removed, similar to the circulatory system in our body. Furthermore, the perfused medium provides shear stress to simulate blood flow through the vasculature and interstitial flow through the extra cellular matrix (ECM) in the hydrogel. Moreover, continuous medium perfusion enables temporal control for treatments and collection of supernatants in a precise time window. The peristaltic pump can simultaneously perfuse 16 chips, thereby allowing for parallelization of multiple conditions in an automatized and reproducible manner. The chips are cultured for 5 days before any analysis or treatments are performed. During the first 2 days of culture the medium is supplemented with 50 ng mL^−1^ vascular endothelial growth factor (VEGF). The following days are cultured with unsupplemented endothelial growth medium.

### 2.2 PDLCs and Continuous Medium Perfusion Improve Network Formation of HUVECs

**Figure 3:**
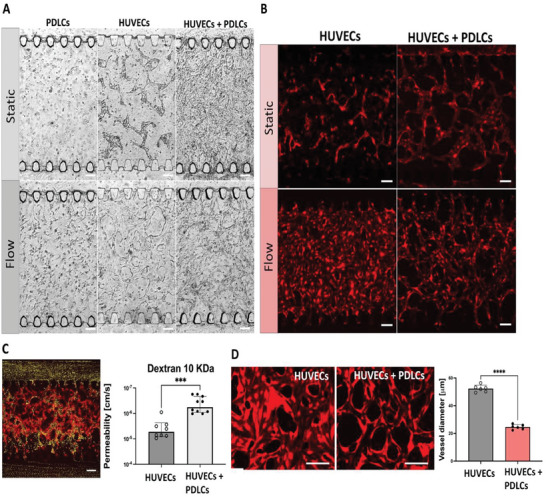
Comparison of different culture conditions and characterization of morphology, perfusability and permeability. A) Bright‐field images of PDLCs, HUVECs and the co‐culture of them two in the central chamber after six days of static and perfused conditions respectively. B) Fluorescence images of vascular network formed by red fluorescent protein (RFP) producing HUVECs in the central chamber under static and perfused conditions respectively. PDLCs are not labeled. C) Analysis of the vessel's permeability after perfused culture conditions using a leakage assay with dextran. The permeability of the vessels is significantly decreased, when HUVECs are co‐cultured with PDLCs, suggesting that the endothelial barrier is stronger. Data is presented as mean ± SD (^***^ *p* = 0.001, *n* = 8 and 10). Visualization of the perfusability with a fluorescent bead suspension (diameter 1 µm) supplied via the side channel of HUVECs and PDLCs co‐culture after perfused cultivation conditions. D) Fluorescence images of RFP HUVECs monoculture and RFP HUVECs‐PDLCs co‐culture under medium perfused culture condition. Vessel diameter measured for the RFP HUVECs monoculture is significantly larger than for the co‐culture of RFP HUVECs and PDLCs. Data presented as mean ± SD (^****^ *p* < 0.0001, *n* = 6). Scalebars: 100 µm.

After 5 days of static culture, with daily exchange of medium through pipette tips, spontaneous network formation occurred in the co‐culture of PDLCs and HUVECs. As a control, a monoculture of HUVECs did not form a continuous network (**Figure** **3**A,B). This observation suggests that PDLCs have an impact on vessel formation, which has previously been showed for pericytes^[^ ^38^, ^39^ ^]^ and fibroblasts.^[^ ^39^, ^40^ ^]^

To make our PDL model more in vivo‐like, we introduce shear stress by perfusing the chips with medium at a flow rate of 60 µL h^−1^ using a peristaltic pump. During continuous medium perfusion, the network formation was improved for HUVECs alone as well as the co‐cultures (Figure 3A,B). However, monocultures of HUVECs had low reproducibility during both static and medium perfused cultivation, where they sometimes did not form networks at all. Furthermore, HUVECs in monoculture formed a monolayer‐like network and were less stable and started to degrade compared to the co‐culture (Figure 3A). Moreover, the co‐culture resulted in smaller vessel diameters compared to HUVECs monoculture (Figure 3D). This has previously been observed for cell types such as pericytes^[^ ^39^ ^]^ that are affecting the morphology of vascular networks. Furthermore, we observed that monocultures of HUVECs tend to overgrow (Figure S3, Supporting Information), a phenomenon previously mentioned as hyperplasia.^[^ ^41^, ^42^ ^]^ These findings suggest that PDLCs have an influence on vessel morphology and maturation.

Given the successful creation and high reproducibility of vascularized tissue formed from continuously perfused co‐cultures, we wanted to characterize this model further. The perfusability was characterized using fluorescent beads and the permeability of the networks were characterized using fluorescent dextran.

### 2.3 Perfusability and Endothelial Barrier Characterization

We characterized the perfusability of the vascular networks. We pipetted 1 µm fluorescent beads into one side channel to observe their transmigration to the opposite side channel through the central vascular network. In medium perfused co‐cultures, the beads were clearly visible in the central chamber inside the vessels while migrating through the network into the opposite side channel (Figure 3C). As a comparison, static co‐cultures were less reproducible in creating perfusable networks (Figure S4, Supporting Information), where the beads did not reach the opposite side channel.

We also compared the barrier functionality of our perfused co‐cultures compared to HUVECs monocultures using a leakage assay with fluorescent dextran (10 kDa) (Figure S5A, Supporting Information), which confirms well‐interconnected micro‐vessel lumens that are also 3D in nature (Figure S5B, Supporting Information). Confocal imaging using a time lapse series was used to capture diffusion of dextran across the endothelial barrier over time. The permeability was calculated in ImageJ^[^ ^43^ ^]^ software following a previously published protocol.^[^ ^38^ ^]^ We observed a lower permeability coefficient in the co‐culture (Figure 3C), suggesting that the PDLCs are increasing the strength of the endothelial barrier. However, both permeability coefficients agreed with the range previously reported in the literature where the coefficient had an exponential range of 10^6^–10^8^ cm s^−1^.^[^ ^40^ ^]^

### 2.4 PDLCs Physically Interact with the Vessels

**Figure 4:**
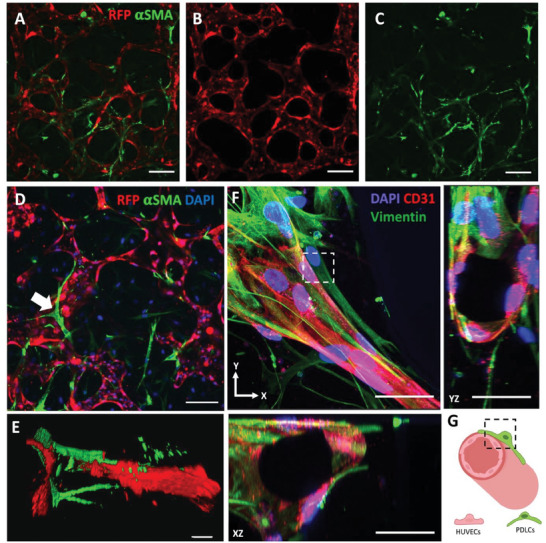
PDLCs undergo striking morphological change in contact with HUVECs and elongate along the blood vessels. A) Fluorescent images of co‐culture of RFP HUVECs (red) and αSMA expressing PDLCs (green) after perfused culture condition presented as an overlay. Individual images of B) HUVECs and C) PDLCs. Scale bar: 100 µm. D) Fluorescence image of RFP HUVECs (red, nucleus blue) and PDLCs (green). PDLCs often exhibit a long shape (see white arrow). Scale bar: 50 µm. E) 3D reconstruction of fluorescent image where PDLCs (green) is wrapping around a branching blood vessel (red). Scale bars 20 µm. F) Cross‐section of micro‐vessel lumen in co‐culture with PDLCs stained with vimentin (green), CD31 (red) and DAPI (blue) visualized in XZ (bottom) and YZ (right) projections. Scale bar: 50 µm. G) Sketch of a cross sections of the blood vessel showing the lumen and the physical contact between HUVECs (red) and PDLCs (green). The box (black) highlights the physical interaction shown in the box (white) in (F).

We observed that PDLCs improved formation and maturation of HUVECs vascular networks. It has previously been suggested that PDLCs have angiogenic properties. However, this was only confirmed from simplistic 2D co‐cultures or using conditioned medium.^[^ ^44^ ^]^ Here, we visualized that PDLCs promoted formation of perfusable 3D vessels. Furthermore, PDLCs displayed striking morphological change in contact with HUVECs. They aligned as elongated sprouts along the blood vessel (**Figure** **4**A–F), clearly visible also in cross sections of the vessel (Figure 4F). A 3D representation of a fluorescent confocal stack showed how PDLCs align in a vessel branching point (Figure 4E). This physical interaction could play a key role in supporting the vessel formation, morphology, and stability. We believe that this explains the more complex and smaller vessel size of the co‐cultured vessels compared to the bulky monolayer like networks resulting from HUVECs alone (Figure 3D).

In contrast to the co‐culture conditions, the PDLCs remained round shaped in static monoculture in absence of HUVECs (Figure 3A), suggesting that HUVECs do have an impact on the cell fate of PDLCs. However, when the monoculture of PDLCs were perfused with medium, the PDLCs also started to form small sprouts (Figure 3A). The morphological change of PDLCs into elongated sprouts is probably due to several factors including increased nutrient access from continuous medium exchange, from growth factors in the endothelial growth medium, shear stress and presence of HUVECs. However, we could show that PDLCs have the ability to differentiate into the osteogenic pathway (Figure S6, Supporting Information). Therefore, it is likely that any of these factors do have an impact on differentiation fate of the PDLCs. In addition, we found that smooth muscle actin (αSMA) is an efficient immunostaining marker to identify PDLCs in co‐culture (as used in Figure 4A–E). The smooth actin properties are associated with vascular smooth muscles environments, which might give a hint toward a potential differentiation path of the PDLCs.

### 2.5 Indirect Co‐Culture of PDLCs Improve Vessel Formation of HUVECs

**Figure 5:**
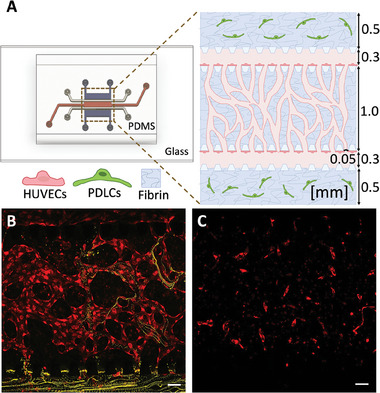
Indirect co‐culture of RFP HUVECs and PDLCs. A) microfluidic device with five separated channels. The right sketch depicts the dimensions with the central part, where vasculature forms, flanked by two medium channels and the outermost chambers filled with PDLCs. B) Fluorescent image of RFP HUVECs (red) in central chamber during static condition resulting in network formation. Furthermore, a fluorescent 1 µm bead suspension is supplied via the side channels. The beads are clearly visible in the central chamber inside vessel, but they do not appear in the opposite side channel indicating that the network is not completely perfusable. C) A fluorescent image of RFP HUVECs (red) in the central chamber during medium perfused culture condition (60 µL h^−1^) resulting in no network formation. Fluorescent images are confocal stacks presented as 2D maximum projections. Scale bars: 100 µm.

Next, we were interested in the role of the PDLCs during vascularization, whether the physical interaction alone is important or if paracrine communication play a crucial role in network formation as well. The paracrine communication between HUVECs and PDLCs was investigated using a five‐channel device. The method, cell count and materials were kept the same as for the three‐channel set up. However, only HUVECs were seeded in the central chamber, and the added flanking chambers separated by media channels, allowing for indirect communication, were seeded with PDLCs (**Figure** **5**A). Indirect co‐culture during static condition resulted in network formation, however, when perfused with 1 µm fluorescent beads they were not completely perfusable (Figure 5B). Indirect co‐culture during perfused conditions (60 µL h^−1^) resulted in no network formation (Figure 5C). This is likely due to the rapid medium exchange preventing sufficient concentrations of secreted factors from PDLCs to reach HUVECs in the central chamber. This indirect model showed that PDLCs do not only physically interact with the vessels, but they also communicate through paracrine pathways by secreting factors to influence vascularization. In vivo, PDLCs are responsible for homeostasis and tissue renewal in a highly dynamic environment influenced by mechanical stress due to mastication. Thus, it is not surprising that these cells have such an active and multifaceted role in forming new tissues.

### 2.6 Detecting Early Immune Response Through Cytokines

**Figure 6:**
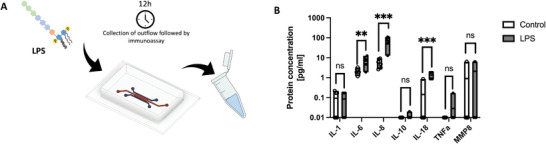
Overview of workflow for emulating inflammation in periodontal ligament model followed by measurement of inflammatory cytokines. A) Medium containing 1 µg mL^−1^ LPS was added to HUVECs and PDLCs co‐cultures achieved from perfused cultivation conditions. The LPS containing medium was perfused at 60 µL h^−1^ and then 120 µL of eluate per chip was collected after 12 h. B) The cytokine content was analyzed in the samples using a sandwiched immunoassay and plotted for IL‐1β, IL‐6, IL‐8, IL‐10, IL‐18, TNF‐α, and MMP‐8. Data presented as mean ± SD (ns = not significant, ^**^ *p* < 0.01, ^***^ *p* < 0.001, *n* = 6).

Periodontitis is an inflammatory disease, manifested as a chronical inflammation of the soft parts of the tooth, that ultimately can lead to tooth loss. The believed cause of oral inflammatory disease is linked to microbial biofilm including virulence factors such as LPS.^[^ ^45^ ^]^ Therefore, we applied the PDL‐on‐chip as a tool to model inflammatory response from LPS in periodontal tissue. After day 5, mature vascular networks (HUVECs and PDLCs co‐cultures, perfused culture conditions) were continuously perfused with endothelial growth medium supplemented with 1 µg mL^−1^ LPS at 60 µL h^−1^ for 12 h. Thereafter, the supernatant was collected and measured for a panel of inflammatory cytokines (Interleukin 1β (IL‐1β), IL‐6, IL‐8, IL‐10, IL‐18, Tumor necrosis factor α (TNF‐α) and Metallopeptidase 8 (MMP‐8) that are all associated with periodontitis (**Figure** **6**A–B).^[^ ^46^ ^]^ Supernatant collected without LPS treatment was used as a control. LPS treatment showed significant upregulation of IL‐6, IL‐8, and IL‐18. Other measured cytokines that was not significantly upregulated were IL‐1β, IL‐10, TNF‐α, and MMP‐8 (Figure 6B).

### 2.7 Quantifying Inflammatory‐ and ECM Markers in Non‐inflamed and Inflamed Tissues

The ECM is a mix of many different macromolecules making up the inter cellular substance of tissues. It plays a key role in regulating cell functions and their fate, by providing adhesion of cells and binding of soluble mediators such as cytokines. There are many factors affecting the ECM composition, including inherited disorders, metabolic disorders, inflammatory disease, or drug‐induced alterations. Up to date there are extensive histological information about periodontal ligaments available. However, the understanding of periodontal ligaments in the context of inflammation or in proliferative or degenerative diseases is still scarce.^[^ ^47^ ^]^ Therefore, we are interested to use our model to quantify relevant ECM and inflammatory markers during inflammation. Collagen is the most abundant ECM protein in PDLs. Other ECM components are laminin, fibronectin, and elastin. In this study, we focus on laminin, collagen I and IV as well as the inflammatory markers E‐selectin and intercellular adhesion molecule 1 (ICAM)−1. Both E‐selectin and ICAM‐1 are receptor proteins expressed on the endothelium early in inflammation to attract immune cells to the inflamed site. We chose these markers due to their abundancy or relevance for inflammatory response.

**Figure 7:**
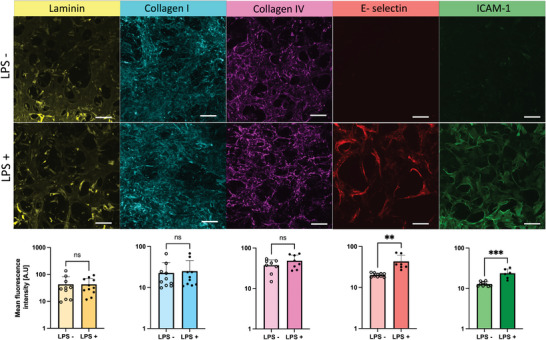
Inflammatory markers and ECM proteins were quantified using immunohistochemistry on non‐inflamed (LPS ‐) and inflamed (LPS +) tissues. Fluorescent images were created through a 2D maximum intensity projection of a 70 µm thick tissue where the mean fluorescence intensity was measured for at least four different ROIs per chip. Each data point represents one individual chip. Data presented as mean ± SD (ns = not significant, ^**^ *p* < 0.01, ^***^ *p* < 0.001, *n* = 10). Scale bars: 100 µm.

The ECM and inflammatory markers were quantified after 12 h of LPS exposure using immunohistochemistry. Confocal imaging was used to acquire 70 µm tissue sections across all samples. The 3D stacks were subsequently processed in ImageJ into a 2D maximum projection of which the mean fluorescence intensity was measured. The inflammatory markers E‐selectin and ICAM‐1 were both clearly upregulated after 12 h of LPS, which was clearly visible in the fluorescence images (**Figure** **7**). Analysis of the fluorescence intensity confirmed the significant increase of E‐selectin and ICAM‐1 receptor proteins. In contrast, the ECM proteins, collagen I, IV and laminin did not show any significant change. Furthermore, we performed second harmonic imaging, which is a label‐free imaging method to study structural integrity of collagen, which did not show any difference in collagen structure or indication of collagen degradation (Figure S7, Supporting Information).

The significant upregulation of endothelial inflammatory markers confirms the response of the endothelium to inflammation. In addition, we repeated the leakage assay with dextran to measure permeability of LPS treated vascular networks where we observed an increased diffusion across the endothelial barrier during inflammation (Figure S8, Supporting Information). Hence, we would like to emphasize the importance of implementing the vascular component in disease models, also in dental models. Overall, the quantification of ECM and inflammatory markers showed that inflammation indeed can be simulated in our model and give a first insight in how the soft highly vascularized tissue in PDL respond to inflammation.

## 3 Discussion

Oral health is tightly linked with the wellness, quality of life and overall health status of individuals. Over 3.5 billion individuals are affected by oral diseases that disturb important physiological functions such as mastication and speech.^[^ ^48^, ^49^ ^]^ Oral diseases often affect the function of other vital organs of the human body and therefore oral pathology is frequently associated with systemic diseases, such as cardiovascular diseases, arthritis, diabetes, pneumonia, obesity, cancers and premature birth. Periodontitis is often linked to the systemic diseases and affects ≈10% of the world's population.^[^ ^11^ ^]^

However, despite the importance of oral health, we are still lacking models allowing the thorough study of the various oral and dental diseases. Microfluidics‐based technology has been proven to be tremendously beneficial for recapitulating the microenvironment of a variety of specialized cells and tissues.^[^ ^16^, ^50^ ^]^ Although seldom dental‐oriented microfluidic approaches have been applied during the last decade in the dental field,^[^ ^24^, ^26^ ^]^ these technologies still remain vastly unexplored in most of the dental disciplines. Microfluidic “organ‐on‐chip” devices constitute precious model systems for the study of many vital organs, and thus constitute important tools in the medical field.^[^ ^51^ ^]^ In the last few years, “organ‐on‐chip” devices started to get an increasing interest for the study of oral and dental health matters. For example, up to date, models for studying either the dentine‐pulp complex and integration of biomaterials, or the host‐microbiome interactions have mainly been explored.^[^ ^29^, ^31^, ^32^ ^]^

The “PDL‐on‐chip” microfluidic device that we developed here is a 3D model that faithfully recapitulates the complexity of a vascularized periodontal ligaments and is relevant for studying periodontal tissues’ physiopathology. Furthermore, our model system sheds light upon in vitro periodontal ligament cells (PDLCs) function and behavior, which can be easily observed due to the unique 3D environment and the flexible design enabled by microfluidics.

Since vasculature is crucial for the vitality and function of most tissues and organs of the human body, “vasculature‐on‐chip” has emerged as a subcategory in “organ‐on‐chips”.^[^ ^52^ ^]^ Great advances have been achieved where endothelial cells have been co‐cultured with various cell types, such as pericytes^[^ ^38^ ^]^ and fibroblasts,^[^ ^53^ ^]^ which are known to play critical roles in blood vessel stabilization and maturation.^[^ ^54^, ^55^ ^]^ These models have shown improved functionality, including barrier function and enhanced angiogenesis, compared to models where the endothelium was cultured alone.^[^ ^53^ ^]^ Interestingly, we show in this 3D “PDL‐on‐chip” in vitro model that PDLCs have a similar function on vascular stabilization and maturation. We created the optimal conditions for the co‐cultures of PDLCs and endothelial cells to generate a perfusable vasculature. Due to the 3D structure of the vasculature, we could reveal the cellular interactions in a more sophisticated manner than was previously showed for these cell types, where PDLCs form elongated sprouts along the blood vessels like pericytes. In addition to the physical organization, PDLCs demonstrated an angiogenic potential that allows their use for regenerative medicine purposes. Furthermore, PDLCs have great clinical potential due to their increased accessibility and do not require any invasive steps.^[^ ^56^ ^]^

We showed that PDLCs have an angiogenic impact on endothelial cells through a paracrine communication. PDLCs and HUVECs were cultured in a five‐channel system in fibrin hydrogels separated from each other by a medium channel, and the HUVECs formed a vascular network under static conditions. However, when cultured under perfused condition (60 µL h^−1^), no networks were formed. This is probably due to the rapid medium exchange which “washes out” and thus prevents cues to diffuse from the PDLCs compartment across the separating medium channel into the HUVECs compartment. It is likely that the indirect co‐culture is strongly dependent on the cell concentration of PDLCs. Higher cell concentration allows for accumulation of a higher concentration of cues that could potentially overcome the rapid medium exchange in the separating channel during perfusion and thereby reach the HUVECs containing chamber and have an impact on vessel formation.

In this work and in accordance with other studies on endothelial cells^[^ ^57^, ^58^ ^]^ and PDLSC spheroids,^[^ ^28^ ^]^ we also showed that flow and shear stress had an impact on the vasculature formation, where the perfused culture condition resulted in a mature and perfusable network. In vivo, cells are exposed to many types of shear stresses, including blood flow and interstitial flow.^[^ ^59^ ^]^ The flow rate of 60 µL h^−1^ corresponding to 0.33 dyne cm^−2^ in our study is, however, low compared to shear stresses found in vivo (5–58 dyne cm^−2^ in blood vessels),^[^ ^60^ ^]^ because we had to find a compromise between a high flow rate and hydrogel stability, as well a high medium consumption.

Once this perfusable vascular “PDL‐on‐chip” model was established, we used it for studying several parameters involved in inflammatory diseases. Culture medium supplemented with LPS endotoxins resulted in significant upregulation of the inflammatory markers IL‐6, IL‐8, and IL‐18. IL‐6 and IL‐8 are both associated with periodontitis.^[^ ^61^ ^]^ Together with molecules such as LPS or TNFα, IL‐6 is activating the inflammatory response in endothelial cells.^[^ ^62^ ^]^ PDLCs have been reported in previous studies to express IL‐6 and IL‐8 upon bacterial challenge,^[^ ^63^ ^]^ indicating that the PDLCs in our study is also likely to contribute to the upregulation of these inflammatory cytokines to initiate the innate immune response.

During inflammation, the local expression of chemotactic cytokines and adhesion molecules such as E‐selectin and ICAM‐1 within the endothelium are key to recruit immune cells such as neutrophiles.^[^ ^62^ ^]^ It has been showed that ICAM‐1 and IL‐8 are expressed in an increased gradient toward the inflamed gingival tissue. Expression of these molecules enables the transportation of neutrophiles from the highly vascularized periodontium to the gingiva sulcus to reach the site exposed to bacterial challenge.^[^ ^64^ ^]^ The significant upregulation of E‐selectin and ICAM‐1 that was observed in our model upon addition of LPS endotoxins, together with IL‐6 and IL‐8 upregulation, confirms that the “PDL‐on‐chip” can recapitulate the innate immune response found in vivo during periodontitis.

LPS was used in this study to induce inflammation, and we detected inflammatory cytokines and upregulation of inflammatory tissue markers. The expression of ECM proteins was analyzed in both physiological and pathological tissues. Despite seeding the cells in a fibrin hydrogel, we could detect presence of the major ECM proteins constituting the periodontal ligaments in vivo including collagen I and IV and laminin.^[^ ^47^ ^]^ This means that the cells are able to deposit new matrix proteins and remodel their microenvironment. It has been reported that collagen breaks down in periodontium during periodontitis in vivo.^[^ ^65^ ^]^ However, we did not observe any significant change in type I collagen, type IV collagen and laminin expression, as well as no signs of collagen degradation during LPS‐induced inflammation. Even though LPS represent an excellent proof‐of‐concept, it is still a simplification compared to in vivo infections since the current model lack live bacteria and immune cells.

In previous studies on bacterial infections on dental tissue models, a transwell system was used as a vascularized gingival connective tissue model to study biofilm formation.^[^ ^66^ ^]^ Makkar et al. also studied bacterial infection in a gingival crevice‐on‐chip where they controlled bacterial colonization with different flow regimes and studied inflammatory cytokines of gingival tissue.^[^ ^32^ ^]^ Even though live bacteria offer a more realistic infection, experiments with bacteria have many challenges to find optimal culture conditions including medium condition and determine adequate infection time and inoculation concentration. In contrast, LPS induced inflammation is very controlled and reproducible and can be compared to other studies.

Overall, our findings indicate the importance of vasculature in disease progression and thereby emphasize the importance of implementing a vascular component in all dental tissue models. In future work, to capture the full complexity and functionality of the periodontal ligaments, it is envisioned to incorporate a porous hard tissue material together with the soft vascularized tissue as well as adding innervation and mechanical stimulation to recapitulate the impact from chewing. However, the vascular network generated in our “PDL‐on‐chip” model can be used as a basis to implement into microfluidic models of other specific dental tissues. The present model is a first cornerstone in creating more complex and sophisticated in vitro microfluidic models for dental tissues.

Furthermore, this model is suitable for many additional applications. The presented model offers advantages for perfusion studies in the microvasculature, for instance of different immune cells to study extravasion across the endothelial barriers similar to the work by Hirth et al.^[^ ^58^ ^]^ and Chen at el.^[^ ^40^ ^]^ Similarly, perfusion studies can be performed with bacteria to study oral sepsis. Future efforts will focus on incorporating additional cell types, such as immune cells, bacteria, and neurons to realistically emulate the complexity of cellular and molecular interactions that occur during periodontitis.

## 4 Conclusion

“PDL‐on‐chip” represents a physiologically relevant 3D microfluidic model of the highly vascularized soft periodontal ligament tissue. This device allows the simulation of early stages of inflammatory diseases, such as periodontitis. PDLCs have an impact on vascular formation and maturation, resulting in the generation of perfusable vascular networks. “PDL‐on‐chip” allowed the close interactions between the two cell populations, where PDLCs behave similarly to pericytes by forming elongated sprouts that surround the developing vessels. Early‐stage inflammatory events were also successfully simulated, since LPS‐induced inflammation caused a significant upregulation of inflammatory cytokines. It is becoming obvious that there is necessity for more efficient microfluidic models to study and better understand periodontal diseases, in order to develop new therapeutic strategies and modalities in the future. Although the microfluidic tissue systems like the “PDL‐on‐chip” have limitations with respect to the in vivo tissue, such as the lack continuous blood perfusion and the lack of immune cells, it represents a viable model for certain aspects of dental health.

## 5 Experimental Section

### Cell Culture

The procedure for the collection of anonymized human periodontal cells at the Center of Dental Medicine (ZZM) of the University of Zurich was approved by the Ethic Commission of the Kanton of Zurich (reference number 2012‐0588) and the patients gave their written informed consent. The teeth were extracted by dentists at clinic of Cranio‐Maxillofacial and Oral Surgery Department at the Center of Dental Medicine of the University of Zurich according to previously described protocols.^[^ ^67^ ^]^ In brief, teeth were immediately collected upon extraction and stored in sterile NaCl 0.9% on ice while transferred to the processing laboratory. The periodontium was isolated by scratching the lower two‐thirds of the root with a surgical scalpel into a petri dish filled with sterile, cold Hank's Balanced Salt Solution (HBSS; Thermo Fisher Scientific, Rheinach, Switzerland). The upper‐third of the root was excluded to minimize contamination of the gingival epithelium. Subsequently, the tissue was transferred into a HBSS‐filled falcon tube and centrifuged at 4 °C, 300 g, for 10 min. The tissue was thereafter digested in 10 mL Collagenase P 5 U mL^−1^ (11 213 873 001, Sigma Aldrich, 199 Buchs, Switzerland) for 40 min at 37 °C. Thereafter, samples were disaggregated, filtered through a 70 µm cell strainer and finally resuspended in HBSS + 0.002% Bovine Serum Albumin (BSA; 0163.2, Roth AG, Arlesheim, Switzerland). PDLCs were cultured to 90% confluency using Dulbecco's Modified Eagle Medium/Nutrient Mixture F‐12 (DMEM/F12) Glutamax (Thermo Fischer) medium supplemented with 10% Fetal Bovine Serum (FBS; Gibco) and 1% penicillin/streptomycin (Thermo Fischer). The cultured cells were incubated at 37 °C, 5% CO_2_ and 95% humidity and used up to passage 7. The medium was changed every other day. Red fluorescent protein (RFP) Human umbilical vein endothelial cells (HUVECs) (Angio Proteomie) were cultured in endothelial growth medium (Vasculife, Cell systems) in 0.2% gelatin coated culture flasks in the same manner described for previous cells.

### Fabrication of Master Molds

The master mold was fabricated using standard photolithography methods. In brief, a standard 4‐inch silicon wafer (Si‐Mat) was dehydrated (10 min, 200 °C) and spin coated with SU‐8 2050 at 3250 rpm for 30 s. The photoresist was baked at 65 °C for 60 s followed by 95 °C for 360 s before it was exposed to 160 mJ cm^−2^ at 365 nm on a MA 6 mask aligner (Süss Microtech) through a transparent photomask that carried either the 3‐channel or the 5‐channel design (Selba S.A). After a post‐exposure bake for 60 s at 65 °C and for 360 s at 95 °C, the wafer was developed using mr‐Dev 600 developer for 5 min. The resulting channel heights were 100 µm. Finally, the master mold was surface treated with 1 H, 1H, 2H, 2H‐ perfluoredecyltrichlorosilane (Sigma) overnight to facilitate the removal of polymer cast.

### Manufacturing of the Microfluidic Devices

Polydimethylsiloxane (PDMS, Sylgard 184) was prepared by mixing 10:1 wt% of polymer and curing agent (Sylgard 184). The mixture was poured onto the fabricated master mold, desiccated until all air bubbles were removed and finally cured in an oven at 80 °C for 3 h. The cured PDMS was cut into individual chips. All inlets and outlets were punched with a 1 mm outer diameter biopsy puncher (Miltex). All surfaces were cleaned and protected with scotch tape (Tesa). Cover glasses (24×40 #1.5, Novoglas) was cleaned with isopropanol and dried with air gun and placed on 80 °C hot plate. The prepared cover glass was placed in the plasma cleaner (Harrick Plasma PDC‐ 32G) together with the PDMS chip with structures facing upward and treated at 50 W for 40 s at brought close together to seal the channels. The bonded chips were placed on the hot plate for 10 min and thereafter stored in the oven at 80 °C overnight to recover hydrophobicity. Before used in cell culture, the chips were sterilized in UV for 30 min and stored in a sterile petri dish.

### Cell Culture on Chip and Continuous Flow Experiments (Perfusion Culture)

The cell seeding procedure including parameters such as cell count, fibrin concentration and flow rates were adapted from our previous work.^[^ ^58^ ^]^ All cell types were grown to 90% confluence, washed with PBS (‐) and detached using 0.05% trypsin‐EDTA(Gibco) and incubated at 37 °C, 5% CO_2_, for 5 min. The trypsin was inactivated in 10% FBS supplemented PBS followed by centrifugation at 1500 rpm for 5 min. The supernatant was removed, and the cells were resuspended in 1 mL of their respective culture medium. Subsequently, the cells were counted. The HUVECs (250 000 cells) were mixed with PDLCs (50 000 cells) at a 5:1 ratio in an Eppendorf tube. They were centrifuged and the supernatant was removed. The pellet was resuspended in 15 µL premix of fibrinogen (12 mg mL^−1^; Merck) from human plasma and aprotinin (0.15 U mL^−1^; Merck). Thrombin from human plasma (Merck) was diluted to 10 U mL^−1^ in Vasculife medium and 1.5 µL was added to the resuspended cells to a final concentration of 1 U mL^−1^. Ten microliters cell‐hydrogel mix was immediately injected into the central compartment of the microfluidic device. The device was flipped up and down for 30 s to prevent potential cell sedimentation. Thereafter, the device was placed in a Petri dish containing a smaller PBS‐filled Petri dish to maintain humidity and incubated at 37 °C, 5% CO_2_, for 15 min. Afterward, the side channels were coated by injecting 20 µL fibronectin (0.1 mg mL^−1^, Merck) diluted in Vasculife medium supplemented with VEGF (50 ng mL^−1^, Peprotech). The devices were incubated over night at 37 °C, 5% CO_2_ and 95% humidity. Next day, the side channels were seeded with HUVECs at a cell density of 100 000 cells mL^−1^ in Vasculife medium. One side channel was seeded at the time and tilted 90° to promote cell adhesion on the gel interface while incubated at 37 °C, 5% CO_2_ and 95% for 2 h. Thereafter, the cell seeding was repeated for the opposite side channel. Then, the chips were connected to 5 mL medium‐filled syringe reservoirs (sterile packaged, Omnifix, 5 mL) via tubing (Ismatec, PharMed BPT, 0.25 mm ID). The tubing was connected to the reservoirs via Luer locks and to the chips via metal pins (Figure S1, Supporting Information). The chips were perfused with VEGF‐supplemented Vasculife medium (50 ng mL^−1^, Rec Human VEGF, Gibco) for the two first days, followed by normal Vasculife medium for the remaining days. The chips were constantly perfused with a peristaltic pump (Ismatec IPC‐N, Cole Palmer) at a flow rate of 60 µL h^−1^ (0.33 dyne cm^−2^) in parallel to the hydrogel interface. In static conditions, the medium was exchanged daily with filled 200 µL filtered pipette tips. The 5‐channel device was prepared in the same manner, but in indirect co‐culture the PDLCs were seeded in the flanking compartment instead of the central compartment. Static and perfused culture conditions for the 5‐channel system were performed in the same manner as previously described for the 3‐channel system.

### Characterization of Permeability, Perfusion, and Vessel Diameter

The perfusability and the endothelial barrier were characterized by perfusing fluorescent dextran. Before imaging, all inlets and outlets were replaced with empty pipette tips. The chips were carefully fixed on the microscope using scotch tape. Cascade blue Dextran (10 KDa, Thermo Fisher) (100 µg mL^−1^ in Vasculilfe medium) was injected into one of the side channels and was diffusing through the vessels to the opposite side channel. The chips were imaged using a Nikon Ti2 spinning disk (Yokogawa) confocal microscope (Visitron) with a 10X objective and a time lapse (20 s interval for 10 min). Z‐stacks were acquired with 6 µm step size of the dextran‐filled vasculature to determine the thickness and volume of the vessels. The stacks were processed in ImageJ using the software plugins “trainable Weka Segmentation 3D” and “Macro permeability” to determine morphological parameters including vessel volume, surrounding matrix volume and surface area of vascular network. Finally, the permeability coefficient was calculated using a template according to previously published protocols.^[^ ^39^ ^]^ Each chip was analyzed for several ROIs.

The perfusability was also characterized by supplying a solution of 1 µm fluorescently FITC‐labeled polystyrene beads (diluted 1:1000, Thermo Fischer) in one of the side channels. The images were recorded as described above and processed in ImageJ, where all timepoint images were projected into a 2D image to visualize their movement through the vasculature. Vessel diameter was measured and quantified from fluorescence images using the measuring function of ImageJ. Each chip was analyzed for several ROIs.

### Immunohistochemistry

The chips were fixed using 4% paraformaldehyde (PFA) (Thermo Fisher) for 30 min at room temperature and then rinsed with PBS (‐/‐) (Gibco) three times for 15 min each. Once fixed, the chips were permeabilized with 0.2% triton X‐100 (Merck) in PBS (‐/‐) for 30 min at room temperature. Then the chips were blocked using 10% goat serum (Thermo Fisher), 0.1% triton x in PBS (‐/‐) for at least 1 h at room temperature. Then, the chips were incubated with primary antibodies (1:100 dilution in 0.1% triton, 1.5% BSA in PBS (‐/‐)) overnight. The following days the chips were washed three times with the antibody dilution buffer each for 15 min. Next, the chips were incubated with secondary antibodies (1:500 dilution in PBS (‐/‐), 0.1% triton‐ X and 1.5% BSA) for 1 h at room temperature. The chips were washed with PBS (‐/‐) three times each for 15 min. Before imaging NucBlue (1:1000 in PBS, Thermo Fisher) was added for 30 min and rinsed three times with PBS (‐/‐). Detailed antibodies are listed in **Table** **1**.

**Table 1:** List of antibodies and staining reagents.

Reagents	Host	Dilution	Supplier	Catalog #
*Primary antibodies*				
Anti‐Collagen 1	Rabbit	1:100	Abcam	ABCAM ab34710
Anti‐Laminin	Rabbit	1:100	Abcam	ABCAM ab11575
Anti‐ICAM‐1	Mouse	1:100	R&D systems	BBA3
Anti‐ E‐selectin	Mouse	1:100	Thermo Fisher	14‐0627‐82
*Secondary antibodies*				
Anti‐Rabbit AlexaFluor 488	Goat	1:500	Thermo Fisher	A32731
Anti‐Mouse AlexaFluor 640	Goat	1:500	Thermo Fisher	A32728
*Conjugated antibodies*				
Anti‐αSMA AlexaFluor 640	Mouse	1:100	Invitrogen	50‐9760‐82
Anti‐Hu CD31 (PECAM‐1) FITCH	Mouse	1:100	Invitrogen	11‐0319‐42
Vimentin Monoclonal Antibody (V9) AlexaFluor 647 *Others*	Mouse	1:100	Invitrogen	MA5‐11883‐A647
NucBlue staining (DAPI)	N/A	1:1000	Thermo Fisher	R37605

### Imaging of Cell–Cell Interactions and Vessel Cross Sections

Chips were fixed with 4% PFA for 30 min followed by three washing steps with PBS. Subsequently, the chips were permeabilize for 30 min followed by blocking for 1 h at room temperature. Then chips were stained with DAPI, CD31 and vimentin over night at 4 °C and was next day washed three times with PBS. The chips were imaged with a confocal microscope (Zeiss LSM 980 Airyscan 2) where the images were subsequently airyscan processed. Z‐stacks were acquired with a 32X objective (zoom 1.3), 0.5 µm stepping size and a thickness of 60 µm. The stacks were further processed in Imaris software version 9.5 to create cross‐sections and 3D constructions of the vessels.

### Detection of Inflammatory Cytokines

After mature vascular networks were formed on day 6, 120 µL supernatant was collected from each of the perfused chips as a control. Then, the chips were perfused with LPS from *Escherichia coli* 026:B6 (Invitrogen, 00‐4976‐93) (1 µg mL^−1^ diluted in Vasculife medium) at 60 µL h^−1^ for 12 h followed by collection of 120 µL supernatant from each of the perfused chips. The collected supernatants were immediately centrifuged at 1500 rpm for 5 min and stored in −80 °C until further use. A personalized commercial procartaplex kit (Thermo Fischer) was used to quantify a panel of cytokines (IL‐1β, IL‐6, IL‐8, IL‐10, IL‐18, TNF‐α, and MMP‐8) in the supernatants. The samples and kit were prepared following manufacturer's guidelines. In brief, 50 µL magnetic bead solution was vortexed and added to each well in a 96 well plate. The magnetic beads were fixed with a magnet and the liquid was removed. The beads were washed and 50 µL of standards and samples were added to different wells. The well plate was sealed and left to shake for 30 min at room temperature and left at 4 °C overnight. Following, the plate was washed two times and 25 µL of detection antibody wash added. The plate was again sealed and incubated with shaking for 30 min in the dark. The beads were again washed two times and 50 µL streptavidin‐PE was added. Another round of incubation and washing was performed again. Then, amplification reagents 1 and 2, respectively, were added, each followed by 30 min incubation with shaking in the dark. Finally, the beads were washed two times and resuspended in 120 µL reading buffer and data were acquired on Magpix (Luminex).

### Characterization of ECM and Inflammatory Markers

Chips that were treated or not treated with LPS for 12 h were fixed and stained with ECM or inflammatory specific markers (laminin, collagen I, collagen IV, E‐selectin and ICAM‐1). The chips were imaged with a Nikon Ti2 spinning disk (Yokogawa) confocal microscope (Visitron) with a tissue thickness of 70 µm and 2 µm stepping size. The image settings were kept constant for each marker to be imaged. Each chip was imaged with a 20X objective in four no‐overlapping areas to avoid photobleaching. The image stacks were further processed in ImageJ where each stack was projected into 2D using a maximum projection function to take the full tissue volume into account. The mean intensity was measured, and minimum intensity was subtracted. The mean values of each chip were plotted to compare treated or nontreated conditions.

### Statistical Analysis

All experiments were performed as at least biological triplicates and at least three regions per device were used for data and image analysis. All data was tested for normality before analyzed using independent *t*‐test. Nonnormal data were tested using the Mann–Whitney U test. Data are presented as mean ± standard deviation (SD). Plotting and statistical analysis of data was performed using GraphPad prism 9.5.1 for Mac (GrapPad Software, San Diego, California, USA). Significant differences were considered if *p* < 0.05.

## Conflict of Interest

The authors declare no conflict of interest.

## Author Contributions

P.S.D. and T.A.M. conceived the concept. P.S.D. and S.S. designed the study. S.S. performed the experiments and analyzed data. E.H. designed the microfluidic device. P.S.D., T.A.M., and S.S. wrote the manuscript, which all approved.

## Supporting information



Supporting Information

## Data Availability

The data that support the findings of this study are available from the corresponding author upon reasonable request.
