# Supplementary material for: “Periodontal ligament‐on‐chip” as a Novel Tool for Studies on the Physiology and Pathology of Periodontal Tissues
Source: Adv Healthc Mater. 2024 Sep 16;13(32):2303942. doi: 10.1002/adhm.202303942 (PMC11670280; doi:10.1002/adhm.202303942)
Supplement: Supplementary file 1 — Supporting Information [file ADHM-13-0-s001.pdf]

ADVANCED HEALTHCARE MATERIALS

Supporting Information

for *Adv. Healthcare Mater.*, DOI 10.1002/adhm.202303942

"Periodontal ligament-on-chip" as a Novel Tool for Studies on the Physiology and Pathology of Periodontal Tissues

*Sara Svanberg, Elisabeth Hirth, Thimios A. Mitsiadis and Petra S. Dittrich**

Supporting Information

“Periodontal ligament-on-a-chip” as a novel tool for studies on the physiology and pathology of periodontal tissues

*Sara Svanberg, Elisabeth Hirth, Thimios A. Mitsiadis and Petra S. Dittrich**

Methods

Osteogenic differentiation: Cells were cultured and differentiated according to manufacturer's instructions. In brief, PDLCs were cultured in a 12 well-plate until 70% confluency in DMEM F12 Glutamax medium supplemented with 10% FBS and 1 % Penicillin at 37°C, 5% CO_2. Then the medium was replaced by pre-warmed Complete Osteogenesis Differentiation Medium (StemPro osteogenesis differentiation kit, Gibco) for 21 days where the medium was refreshed every 3-4 days. Subsequently, the cells were stained for Alzarin Red S staining following provider's protocol. Briefly, media was removed and rinsed with DPBS before being fixed with 4% PFA for 30 minutes. After fixation, wells were rinsed with distilled water and a 2% Alzarin S staining solution (Sigma Aldrich) (pH 4.2) was added for 3 minutes. The wells were finally again rinsed three times with distilled water before imaging. Then color bright-field images were acquired using a plate reader (BioTek Cytation 5) to capture the mineralization. The Alzarin S Red staining showed that PDLCs have formed red-stained mineralization. Scale bar: 1 mm.

Second harmonic imaging: Second harmonic imaging was performed on HUVECs and PDLSCs co-cultures that were cultured under perfused condition and subsequently treated with LPS as described in the main method section. The cells were kept alive and were not fixed prior to imaging. Stacks (35 µm thick, stepping size 2 µm) were acquired with confocal microscopy (Zeiss LSM 980) with a 40X objective, excitation 850 nm and with 30% power. Subsequently, the images were processed in ImageJ with a sum projection function.

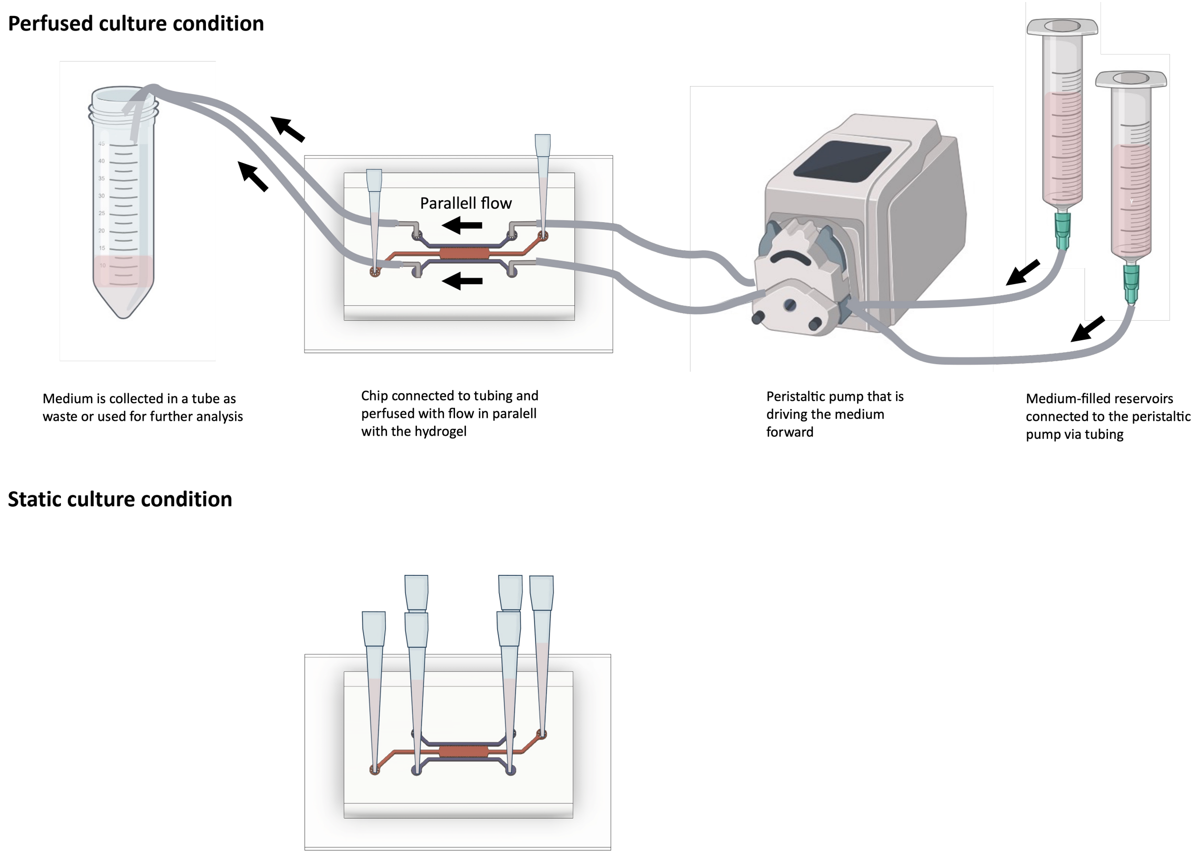

Figure S1. Sketch of the perfused culture condition and static condition, respectively. The perfused culture set up has medium-filled syringes as reservoirs connected to a peristaltic pump that pushes the medium through the chip in direction of the arrows, introducing a flow and shear stress parallel to the hydrogel. Tubing is also connected to the chip for collecting the medium and subsequent analysis of inflammatory cytokines in the supernatant. The static condition is cultured by daily exchanging medium-filled 200 µl pipette tips. This figure has partly been created with BioRender.com.

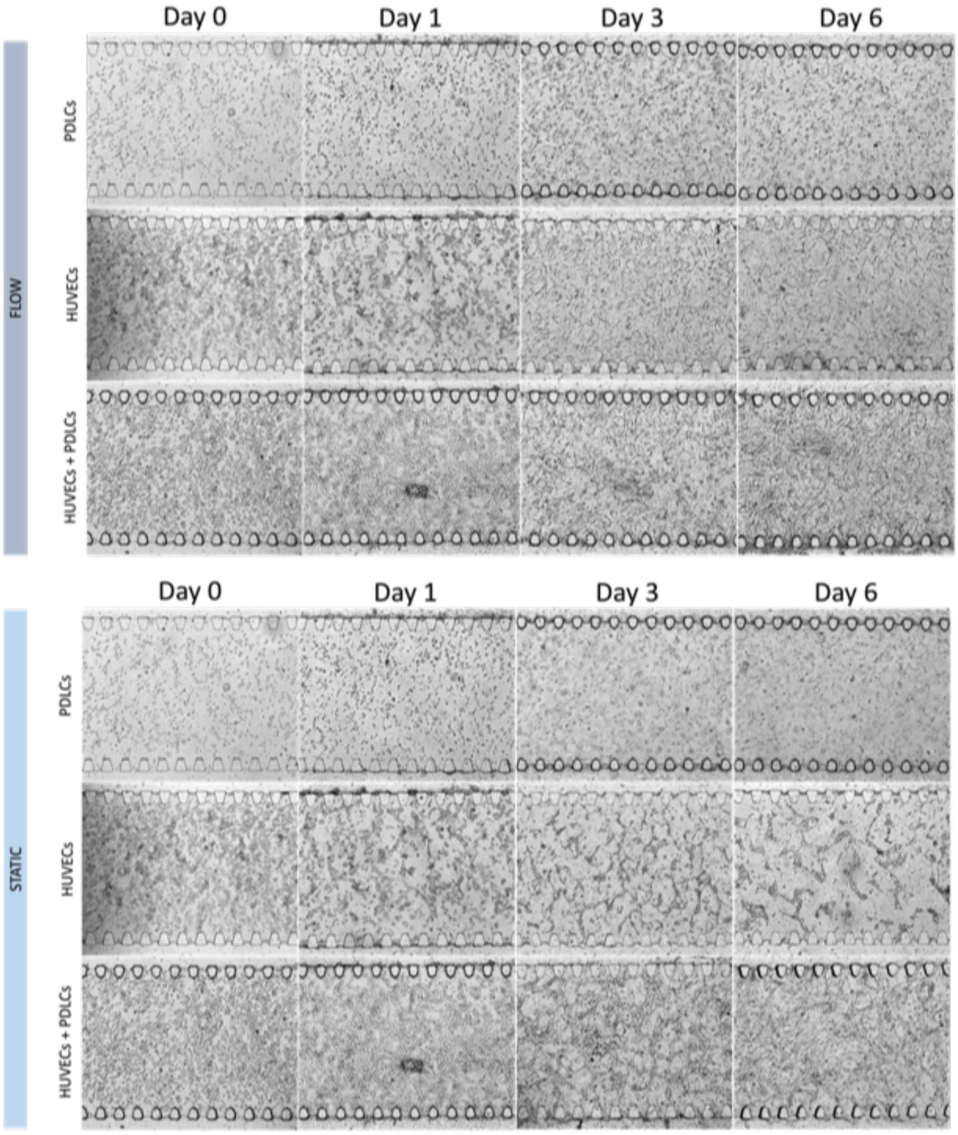

Figure S2. Bright-field images monitoring the daily growth of mono- and co-cultures of HUVECs and PDLCs respectively cultured with either static condition or perfused with medium at a flow rate of 60 µl/h for six days. The PDLCs monocultures remain round shaped under static culture condition, whereas they morphologically change and elongate when cultured under perfused conditions. HUVECs do not form continuous networks when cultured under static conditions. When HUVECs are cultured under perfused conditions the networks are improved but are not stable as they start to degrade compared to the co-culture with PDLCs. When HUVECs are co-cultured with PDLCs, networks are formed during both static and perfused conditions. However, under perfused condition the connection to the side channels formed better. Cells were seeded and cultured in the devices as described in the main paper.

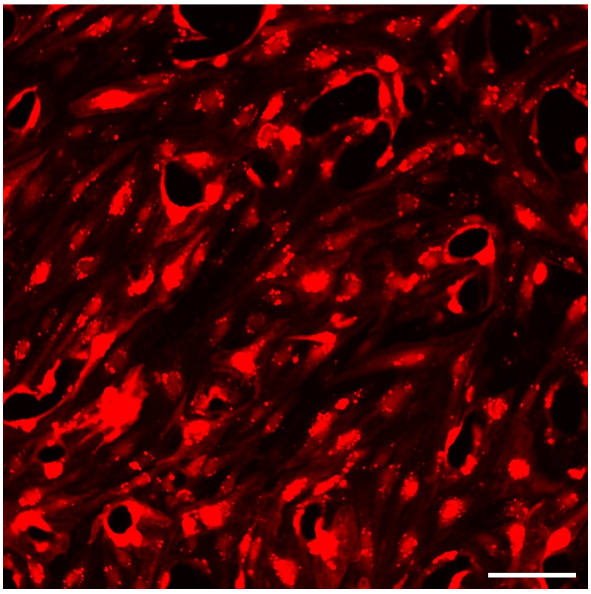

Figure S3. Fluorescent image (20X) of monoculture of RFP producing- HUVECs (red) cultured with medium perfusion at a flow rate of 60 µl/h for 6 days resulted in formation of a monolayer and hyperplasia. The presented image is a 2D maximum project of a confocal stack created in ImageJ. The device was seeded, cultured and imaged as described in main paper. Scale bar: 100 µm.

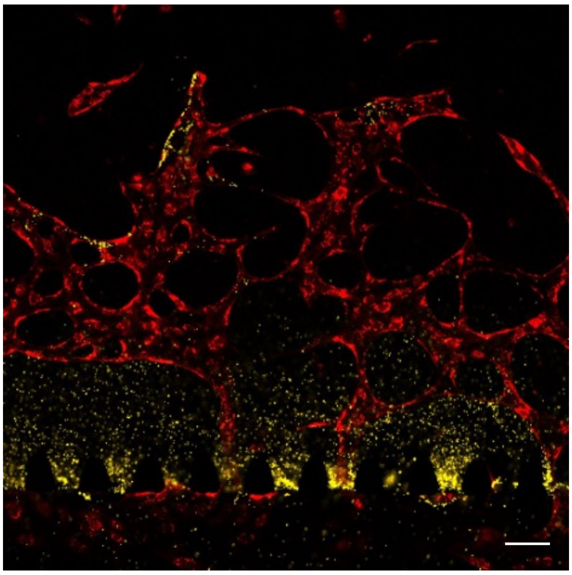

Figure S4. Fluorescent image of static co-culture of RFP-producing HUVECs (red) and PDLCs (not labeled) perfused with 1 µm-large fluorescent beads (yellow) by pipetting the solution into only one of the side channels. The fluorescent beads do not enter the microvasculature and do not migrate to the opposite side channels, indicating that this condition was not perfusable. The confocal stack is presented as a 2D maximum projection created in ImageJ. The device was seeded, cultured and imaged as described in the main paper. *Scale bar: 100 µm.*

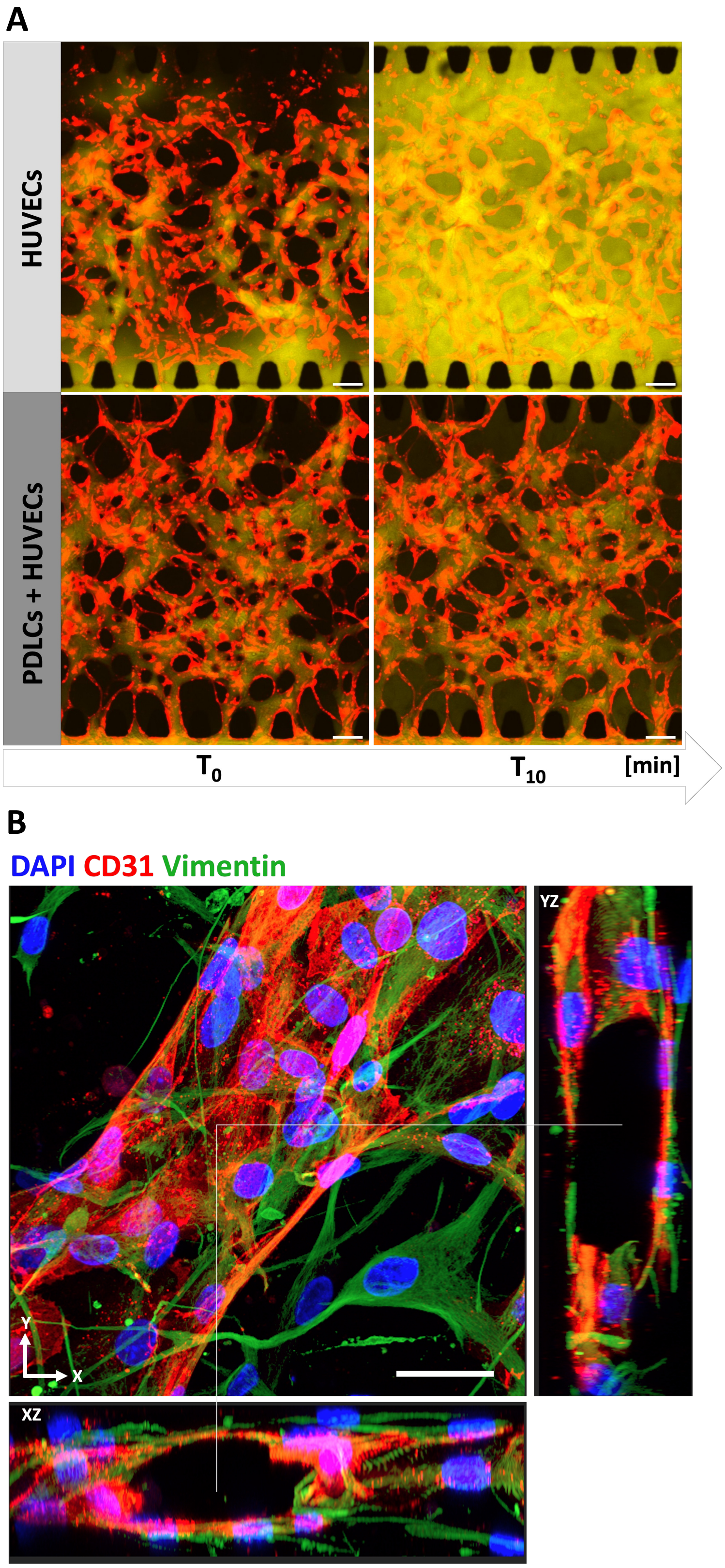

Figure S5. A) Fluorescence images of RFP-producing HUVECs vascular networks (red) with or without presence of PDLCs (unlabeled) filled with fluorescent Cascade blue dextran (10 KDa, 100 ug/ml in Vasculife medium) after 0 and 10 minutes, respectively. After 10 minutes,

the monoculture network show leakage of dye outside the vessels compared to the co-culture, indicating that PDLCs are improving the endothelial barrier. The presented images are 2D maximum projections of the z-stacks processed in ImageJ. The cells were seeded, cultured and imaged as described in the main paper. Scale bars: 100 μm. B) Cross-section of micro-vessel lumen in co-culture with PDLCs stained with vimentin (green), CD31 (red) and DAPI (blue) visualized in XZ (bottom) and YZ (right) projections. Scale bar: 40 μm.

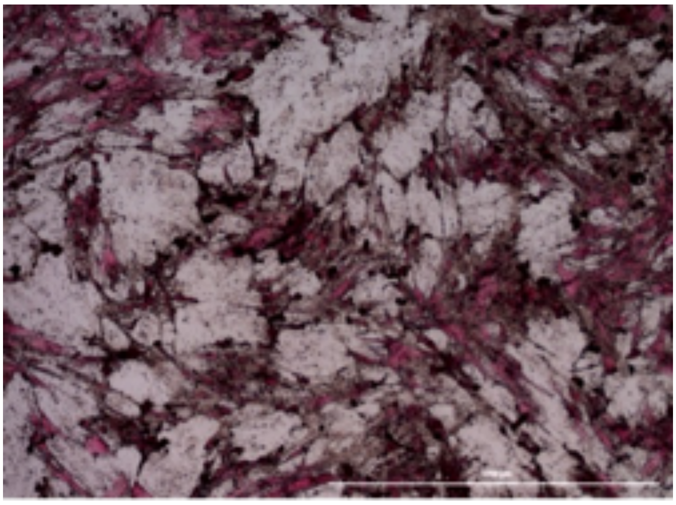

Figure S6. Bright-field image of PDLCs after 21 days of osteogenic differentiation stained with Alzarin S Red staining solution. The red color shows the mineralization. Scale bar: 1 mm.

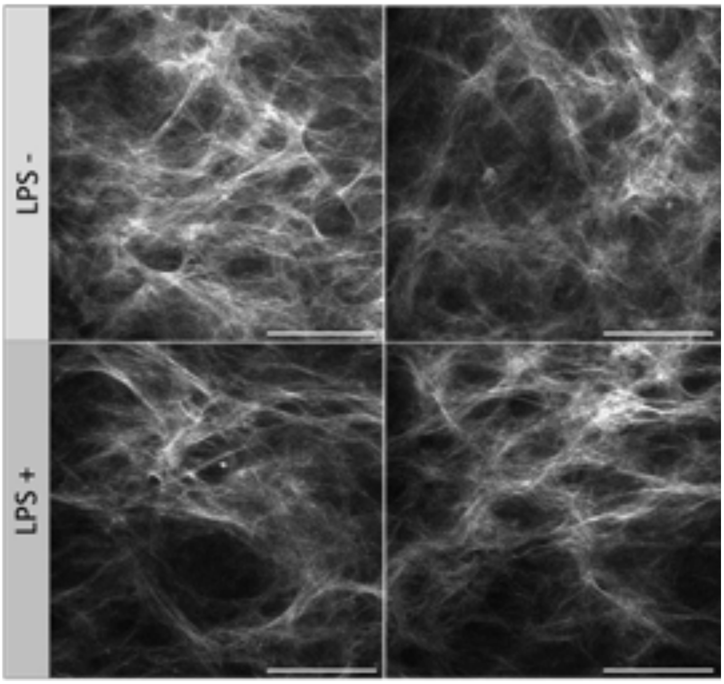

Figure S7. Second harmonic images of collagen in uninflamed (LPS -) and inflamed (LPS+) co-cultures of HUVECs and PDLCs cultured with medium perfusion at 60 µl/h for 6 days. Confocal stacks were acquired and subsequently processed in ImageJ with a sum projection function. The devices were seeded, cultured, and inflamed as described in the main paper. Scale bars: 100 µm.

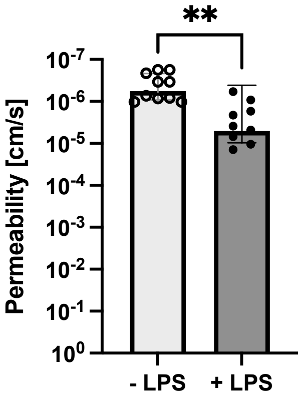

Figure S8. Permeability constant calculated for not inflamed (LPS-) and inflamed (LPS+) vasculature (PDLCs and HUVECs, perfusion culture). Cascade blue dextran (10 KDa, 100 ug/ml in Vasculife medium) were added to the vasculature and was imaged after 0 and 10 minutes respectively. The LPS treated networks showed increased leakage of dextran compared to nontreated networks. (N=3, n= 9 and 10, **p=0.01). Significance level was determined using an unpaired t-test. Data is presented as mean ± SD. The devices were seeded, cultured, inflamed, and imaged as described in the main paper.
